# Alternative first exon splicing regulates subcellular distribution of methionine sulfoxide reductases

**DOI:** 10.1186/1471-2199-7-11

**Published:** 2006-03-16

**Authors:** Hwa-Young Kim, Vadim N Gladyshev

**Affiliations:** 1 Department of Biochemistry, University of Nebraska, Lincoln, Nebraska 68588, USA

## Abstract

**Background:**

Methionine sulfoxide reduction is an important protein repair pathway that protects against oxidative stress, controls protein function and has a role in regulation of aging. There are two enzymes that reduce stereospecifically oxidized methionine residues: MsrA (methionine-*S*-sulfoxide reductase) and MsrB (methionine-*R*-sulfoxide reductase). In many organisms, these enzymes are targeted to various cellular compartments. In mammals, a single MsrA gene is known, however, its product is present in cytosol, nucleus, and mitochondria. In contrast, three mammalian MsrB genes have been identified whose products are located in different cellular compartments.

**Results:**

In the present study, we identified and characterized alternatively spliced forms of mammalian MsrA. In addition to the previously known variant containing an N-terminal mitochondrial signal peptide and distributed between mitochondria and cytosol, a second mouse and human form was detected *in silico*. This form, MsrA(S), was generated using an alternative first exon. MsrA(S) was enzymatically active and was present in cytosol and nucleus in transfected cells, but occurred below detection limits in tested mouse tissues. The third alternative form lacked the active site and could not be functional. In addition, we found that mitochondrial and cytosolic forms of both MsrA and MsrB in *Drosophila *could be generated by alternative first exon splicing.

**Conclusion:**

Our data suggest conservation of alternative splicing to regulate subcellular distribution of methionine sulfoxide reductases.

## Background

The sulfur atom of methionine residues is susceptible to oxidation by reactive oxygen species. Oxidation of methionines may affect protein structure and function and has been implicated in various processes, such as oxidative stress, accelerated aging, and neurodegenerative diseases (reviewed in [1-6]). This oxidation generates a diastereomeric mixture of methionine-*S*-sulfoxide and methionine-*R*-sulfoxide. In contrast to irreversible protein oxidation, such as carbonylation, methionine sulfoxide can be reversibly reduced back to methionine by repair enzymes, methionine sulfoxide reductases. There are two known classes of these enzymes: MsrA (methionine-*S*-sulfoxide reductase) and MsrB (methionine-*R*-sulfoxide reductase). Although the catalytic mechanisms for MsrA and MsrB are similar, these enzymes have different folds.

Most organisms from bacteria to humans encode MsrA and MsrB genes in their genomes, however, some hyperthermophiles lack MsrA, MsrB, or both genes [7]. MsrA and MsrB genes in several bacterial species are clustered with each other and appear to form an operon. In addition, MsrA and MsrB proteins are often linked to each other via direct domain fusion [7,8]. Single MsrA and MsrB genes are present in yeast (e.g., *Saccharomyces cerevisiae*) and many other species (e.g., *Caenorhabditis elegans *and *Drosophila melanogaster*) [7]. On the other hand, multiple genes of MsrA and MsrB have been found in members of the plant kingdom, *Arabidopsis thaliana *[9,10] and *Chlamydomonas reinhardtii *[11].

Human and mouse genomes contain one MsrA and three MsrB genes [12,13]. The three mammalian MsrB products are located in different cellular compartments [13]. MsrB1 (also known as selenoprotein R or selenoprotein X) is a cytosolic and nuclear protein. MsrB2 (also known as CBS-1) is targeted to mitochondria. Human MsrB3 gives rise to two forms generated by alternative first exon splicing, which are targeted to the endoplasmic reticulum (ER) and mitochondria [13], whereas there is no evidence for alternative first exon splicing in the mouse MsrB3 gene [14]. Although only a single MsrA gene is found in mammals [15,16], the corresponding protein is localized in both cytosol and mitochondria [17]. Recently, we reported that the mouse MsrA containing the N-terminal mitochondrial signal peptide is targeted to cytosol, nucleus, and mitochondria [18].

Alternative splicing is viewed as a major mechanism responsible for multiplicity of protein forms in higher eukaryotes [19]. Herein, we have found and characterized a new splice form of MsrA in mouse and human. We also report on alternative splicing of MsrA and MsrB genes in insects. Our data are consistent with the idea that alternative splicing is a conserved mechanism to regulate subcellular distribution of methionine sulfoxide reductases in mammals and other animals.

## Results and discussion

### Alternative splicing variants of human and mouse MsrA

**Figure 1:**
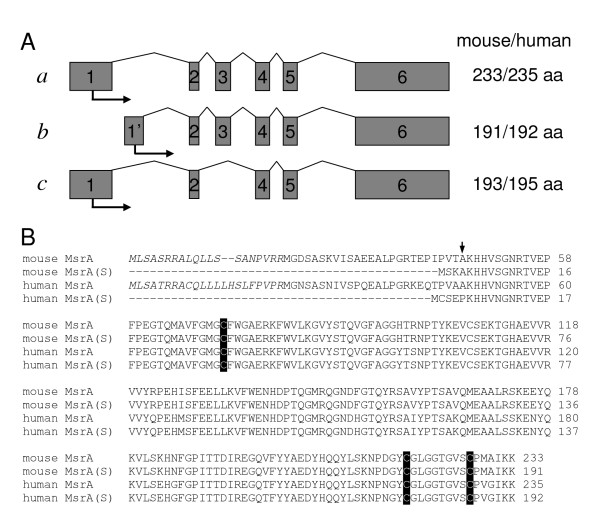
**Splice variants of mouse and human MsrA**. (A) Schematic illustration of genomic structures of alternatively spliced MsrA forms in humans and mice. Translation initiation sites are indicated by arrows. Exons are shown by boxes and are numbered. The number of residues in each of the human and mouse forms is indicated on the right. (B) Alignment of mouse and human mitochondrial MsrAs (the *a *form in A) and MsrA(S)s (the *b *form in A). The mitochondrial signal peptide is indicated in italics. The catalytic cysteine in the GCFWG sequence and two cysteine residues in the last exon are highlighted. The arrow indicates the last amino acid residue of exon 1.

As indicated in the Background, only a single MsrA gene is present in human, mouse, and rat genomes. The 374-kb human and 317-kb mouse MsrA genes consist of 6 exons, which generate a previously known form of the enzyme. This MsrA form contains an N-terminal mitochondrial signal peptide (known as the mitochondrial MsrA form and shown as the *a *form in Figure 1A, [GenBank: mouse, NM_026322; human, NM_012331]). However, its subcellular location is complex. In addition to mitochondria, this protein is localized in cytosol and nucleus [18]. To identify whether additional MsrA forms are present in humans and mice, non-redundant and EST sequence databases were searched using the known MsrA sequences and the identified cDNAs were mapped on the genomic sequences. Several alternatively spliced variants were detected in both humans and mice. The most relevant alternative spliced form was generated by alternative first exon splicing (the *b *form in Figure 1A, [GenBank: mouse, AK018338; human, AY690665]). It was mostly found in brain ESTs, especially in early developmental stages (e.g., GenBank: F06683, H08256, R55838). This alternative variant of mouse MsrA had an ORF of 191 residues (192 residues in humans), of which residues 5–191 corresponded to residues 47–233 of the mitochondrial MsrA form (Figure 1B). This form was designated as MsrA(S). Additional alternative forms were also detected, but these were likely enzymatically inactive. For example, an alternative form devoid of exon 3 was identified (the *c *form in Figure 1A, [GenBank: mouse, BC014738; human, CK819754]). However, this form lacked the active site and it was not detected (based on its lower molecular weight) in several mouse tissues tested and in mouse fibroblast NIH 3T3 cells by western blot analyses (data not shown). An additional alternative form has also been detected, in which exon 6, the last exon, was replaced with a segment of an unknown gene [GenBank: CD 365491]. This form shared the first exon with the mitochondrial MsrA form, but it is likely catalytically inactive due to lack of the last exon which contains two cysteine residues critical for catalysis. Other detected forms contained just some of the MsrA exons.

### MsrA(S) is enzymatically active and localized in cytosol and nucleus

**Figure 2:**
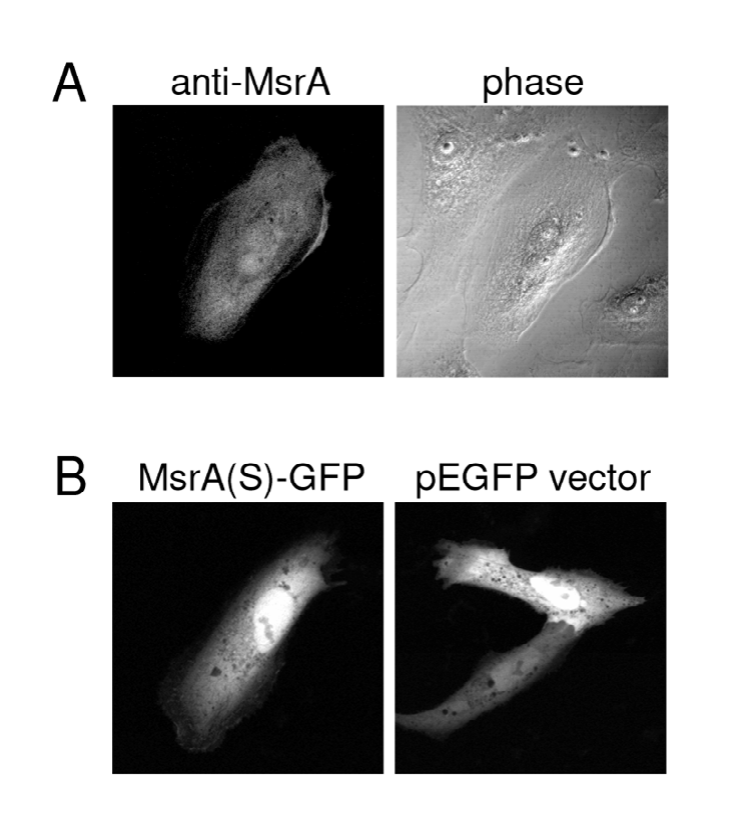
**Subcellular localization of the MsrA(S) form**. (A) The MsrA(S) expression construct (pMsrA(S)ΔGFP) was transfected into monkey kidney CV-1 cells. Transfected cells were stained with anti-MsrA antibodies followed by secondary anti-rabbit Cy5-conjugated antibodies. A set of two images is shown: left panel, immunofluorescence; right panel, phase contrast. (B) Green fluorescence images of CV-1 cells expressing GFP-fused MsrA(S) (left) and control proteins (pEGFP vector, right).

We prepared a recombinant MsrA(S) form and characterized its catalytic activity. In parallel, we measured the activity of the mitochondrial MsrA form for comparison. The specific activity of MsrA(S) was 90 ± 7 nmol/min/mg protein, which is 3-fold lower than that of the mitochondrial MsrA (260 ± 9 nmol/min/mg protein). The K_m _value of MsrA(S) was 1.4 ± 0.3 mM, which is 4-fold higher than that of the mitochondrial MsrA (0.37 ± 0.1 mM). To characterize subcellular localization of MsrA(S) in mammalian cells, we generated an expression construct containing a full-length MsrA(S). The construct, pMsrA(S)ΔGFP, was transfected into CV-1 cells, and MsrA(S) location was determined by immunofluorescence using polyclonal MsrA antibodies. As shown in Figure 2, the protein was located in cytosol and nucleus. The presence of MsrA(S) in these compartments was also confirmed by transfecting cells with a GFP-fusion construct, pMsrA(S)-GFP.

**Figure 3:**
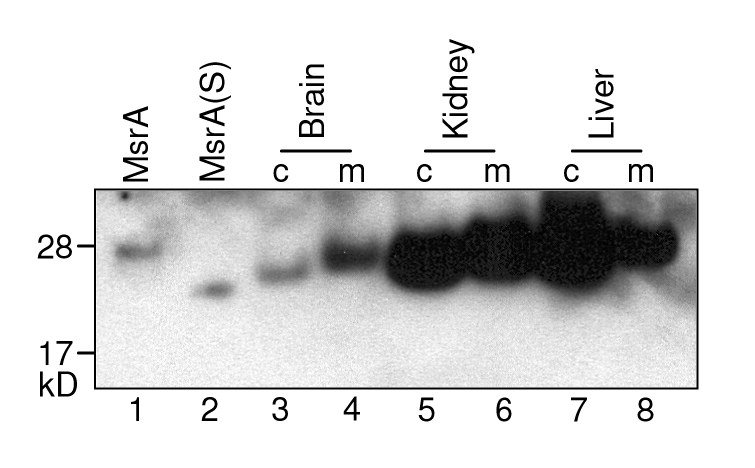
**Western blot analysis of MsrA forms**. Cytosolic and mitochondrial fractions from adult mouse brain, kidney, and liver [18] were used. Equal amounts (30 μg) of proteins were loaded on SDS-PAGE. Lanes 1 and 2, purified recombinant His-tagged mitochondrial MsrA and His-tagged MsrA(S); lanes 3 and 4, brain cytosolic and mitochondrial fractions; lanes 5 and 6, kidney cytosolic and mitochondrial fractions; lanes 7 and 8, liver cytosolic and mitochondrial fractions.

Previously, we demonstrated that MsrA derived from the protein form containing the mitochondrial signal peptide is localized in cytosol and nucleus as well as in mitochondria and proposed that the subcellular localization of MsrA is controlled on a post-translational level [18]. To test whether MsrA(S) can be detected in mouse tissues, we carried out western blot analyses. Based on size comparison with bacterially-expressed His-tagged mitochondrial MsrA and MsrA(S) that served as internal controls, we did not detect the MsrA(S) form in any of the mouse tissues tested (brain, kidney, and liver) (Figure 3). However, the MsrA with the higher molecular weight (derived from the form containing the mitochondrial signal peptide) was easily detected in cytosol and mitochondria of these tissues. As described previously [18], the cytosolic form was slightly smaller than the form found in mitochondria, but both of them were larger than the recombinant MsrA(S). It is likely that the expression level of MsrA(S) is too low to be detected or this form occurs in tissues not tested in the current study. However, this form is clearly detectable in EST databases in various organisms. In addition, a recent review by Hansel *et al*. [12] stated that an alternative form of MsrA mRNA was detected by RT-PCR in human HEK 293 embryonic kidney cells.

### Alternative splicing forms of MsrA and MsrB in drosophila

**Figure 4:**
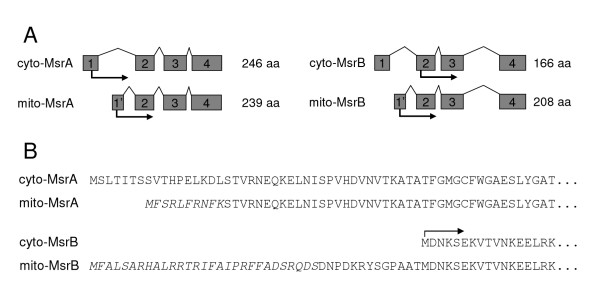
**Alternative splicing of *D. melanogaster *MsrA and MsrB genes**. (A) Genomic structures of *Drosophila *MsrA and MsrB genes. Translation initiation sites are indicated by arrows. Boxes indicate exons, which are numbered. Upper images show organization of the cytosolic forms of MsrA (left) and MsrB (right), respectively, and lower images of the mitochondrial forms of these proteins. (B) N-terminal amino acid sequences of alternatively spliced variants of MsrA and MsrB. Arrow indicates initiation of translation of cytosolic MsrB. However, this methionine is an internal residue in the mitochondrial MsrB form. Mitochondrial signal peptides are shown in italics.

Single MsrA and MsrB genes were identified by BLAST searches in *D. melanogaster*. Known MsrA [GenBank: AF541958] and MsrB [GenBank: AY070627] proteins are thought to be represented by cytosolic forms [20]. We searched for possible additional forms of these proteins in EST and cDNA sequence databases and identified an alternative splicing variant of MsrA [GenBank: AI064299] and MsrB [GenBank: NM_169368]. These forms were generated by alternative first exon splicing. They contained a predicted mitochondrial signal peptide at the N-terminus (Figure 4B) and were predicted to be mitochondrial proteins. The genomic structure consisting of 4 exons and the alternative splicing pattern of MsrA were similar to those of MsrB (Figure 4A). However, as shown in deduced amino acid sequences (Figure 4B), the translation initiation codon of the cytosolic MsrB was located in exon 2.

We suggest that the two alternatively spliced forms of *D. melanogaster *MsrA and MsrB repair oxidized methionine residues in the cytosol and mitochondria, which are the locations that correspond to the occurrences of mammalian MsrA and MsrB in these compartments. We also analyzed additional insect sequences and found that the alternative *D. melanogaster *forms are conserved in *D. yakuba *and *D. pseudoobscura*. In addition, the predicted cytosolic and mitochondrial forms of MsrA were represented by *Glossina *EST sequences. Although further studies on the expression levels of MsrA(S) and occurrence of this protein form in various mouse tissues require more sensitive assays that are currently not available (and the *in vivo *proof of the occurrence of multiple MsrA forms in fruit flies is also needed), it seems remarkable that the same mechanism is used to regulate subcellular distribution of MsrA in such different organisms as fruit flies and mammals, and that in each case, cytosolic and mitochondrial forms are synthesized.

## Conclusion

This study shows that in some animals alternative splicing is a common mechanism to regulate subcellular distribution of methionine sulfoxide reductases, allowing targeting the system for repair of oxidized methionine residues to different compartments.

## Methods

### Preparation of recombinant alternatively spliced form of mouse MsrA

To generate a C-terminal His-tagged MsrA(S) (an alternative form of MsrA), PCR was performed with primers ET-SF (5'-CGCCATATGTCTAAAGCCAAACACCATGTCAGTGG-3') and ET-C (5'-ACACCTCGAGTTTTTTAATGGCCATCGGGC-3') using pET-MsrA(L)-His which encodes a C-terminal His-tagged mitochondrial MsrA [18]. The amplified fragment was cloned into the *Nde*I/*Xho*I sites of pET21b, resulting in pET-MsrA(S). The mitochondrial MsrA and MsrA(S) proteins were expressed in *E. coli *BL21(DE3) cells, purified using TALON metal affinity resin (Clontech) and analyzed for purity by SDS-PAGE. Protein concentration was determined by the Bradford method using bovine serum albumin as a standard.

### Determination of MsrA activity and K_m_

MsrA activity was assayed using dabsylated L-methionine-*S*-sulfoxide as a substrate. A typical reaction mixture (100 μl) for reduction of dabsyl-methionine-*S*-sulfoxide to dabsyl-Met contained 50 mM sodium phosphate (pH 7.5), 50 mM NaCl, 20 mM DTT, 200 μM substrate, and 1–2 μg of purified proteins. The reaction was carried out at 37°C for 30 min, and stopped by adding 200 μl of acetonitrile. The reaction product, dabsyl-Met, was analyzed by HPLC as described previously [20]. For determination of K_m_, 50–800 μM substrate was used. The K_m _values were determined using Lineweaver-Burk plots.

### Constructs for subcellular localization

A GFP-fusion construct was prepared using pEGFP-N1 (Clontech). Full-length MsrA(S) was PCR-cloned into the *Xho*I/*Eco*RI sites of pEGFP-N1, resulting in pMsrA(S)-GFP. To create an MsrA(S)-only expression construct, a full-length PCR fragment of MsrA(S) was cloned into the *Xho*I/*Bsr*GI sites of pEGFP-N1 that replaced the GFP coding sequences. The construct was designated as pMsrA(S)ΔGFP.

### Fluorescence confocal microscopy

Transfections into monkey kidney CV-1 cells were performed using Lipofectamine (Invitrogen). For immunofluorescence staining of MsrA(S), cells were fixed as described previously [18]. MsrA(S) proteins were stained with polyclonal anti-rat MsrA antibodies (kindly provided by Bertrand Friguet), followed by secondary anti-rabbit Cy5-conjugated antibodies (Jackson ImmunoResearch Laboratories). Images were collected using a BioRad MRC1024ES laser scanning microscope.

### Western blotting

Polyclonal antibodies against rat MsrA were used to detect MsrA proteins from mouse tissues and NIH 3T3 cells. Proteins were visualized using chemiluminescent peroxidase substrate (Sigma).

### Searches for alternatively spliced forms of animal MsrA and MsrB

To identify alternatively spliced forms of MsrA and MsrB, BLAST searches were carried out using various MsrA and MsrB sequences against NCBI non-redundant, EST and genomic databases. Only forms represented by at least 2 cDNA sequences were further considered. To determine gene structures, cDNA sequences were aligned with corresponding genomics sequences. Signal sequences were predicted with Signal P.

While this manuscript was under review, a study currently in press reported 5'RACE (5' rapid amplification of cDNA ends) using human retina cDNA and identified the human MsrA(S) form [21]. This finding provides further support for the occurrence of this form. In addition, an additional macaque EST (CJ442767) was included in the EST databases, which suggests the occurrence of an additional mammalian MsrA form with an alternative exon.

## List of abbreviations

ER, endoplasmic reticulum; EST, expressed sequence tag; MsrA, methionine-*S*-sulfoxide reductase; MsrB, methionine-*R*-sulfoxide reductase; ORF, open reading frame

## Authors' contributions

HYK designed the study, performed the experiments, analyzed the data, and wrote the manuscript. VNG conceived the study, participated in its design, analyzed the data, and wrote the manuscript. All authors read and approved the final manuscript.
